# Evaluation of large language models for nursing support in maternal venous thromboembolism care

**DOI:** 10.3389/fpubh.2026.1740254

**Published:** 2026-04-24

**Authors:** Cijuan Li, Yingtao Lin, Deqin Chen, Yuanqing Huang

**Affiliations:** 1 Department of Comprehensive Surgery, Fujian Maternity and Child Health Hospital, College of Clinical Medicine for Obstetrics and Gynecology and Pediatrics, Fujian Medical University, Fuzhou, Fujian, China; 2 Clinical Medical Research Center, Fujian Cancer Hospital, Clinical Oncology School of Fujian Medical University, Fuzhou, Fujian, China; 3 Fujian Maternity and Child Health Hospital, College of Clinical Medicine for Obstetrics and Gynecology and Pediatrics, Fujian Medical University, Fuzhou, Fujian, China

**Keywords:** artificial intelligence, clinical decision support, Delphi consensus, large language models, maternal nursing, nursing informatics, patient education, venous thromboembolism

## Abstract

**Purpose:**

Venous thromboembolism (VTE) is a major cause of maternal morbidity and mortality, and nursing plays a central role in prevention, patient education, and follow-up. Large language models (LLMs) have attracted increasing attention in healthcare; however, their comparative performance in maternal VTE nursing contexts remains insufficiently explored.

**Methods:**

Five representative LLMs—DeepSeek, GPT-4.1, Claude 3.7, Huatuo, and Kimi—were evaluated across six clinical domains (etiology, diagnosis, treatment, prognostic assessment, home care, prevention) and five performance dimensions (accuracy, comprehensibility, logical coherence, reliability, safety). An expert-informed Delphi framework comprising 41 items guided the evaluation. Three nursing experts independently rated each model’s responses, and inter-rater reliability was assessed using Fleiss’s Kappa.

**Results:**

GPT-4.1, Claude 3.7, and DeepSeek demonstrated superior overall performance, particularly in patient education, individualized care planning, and preventive guidance. Huatuo and Kimi showed limitations in treatment and prognostic reasoning. Inter-rater reliability was excellent (Kappa = 0.892).

**Conclusion:**

The findings highlight relative strengths and limitations of different LLMs across nursing-relevant domains in maternal VTE care. While certain models performed better in educational and supportive contexts, the current study does not assess clinical adequacy or readiness for real-world nursing deployment. Future research incorporating patient perspectives and real-world validation is needed to inform the safe and appropriate integration of LLMs into nursing practice.

## 1 Introduction

Venous thromboembolism (VTE), encompassing deep vein thrombosis and pulmonary embolism, is a major cause of maternal morbidity and mortality worldwide. Physiological changes during pregnancy, including hypercoagulability, venous stasis, and vascular injury, markedly increase the risk of VTE during both pregnancy and the postpartum period (1, 2). Early recognition and effective management are essential to improve maternal outcomes. However, clinical decision-making remains challenging due to heterogeneous presentations, and gaps in health education and self-management often limit patients’ ability to prevent and respond to complications (3). From a nursing perspective, these challenges underscore the need for enhanced patient education, follow-up care, and supportive communication throughout the continuum of maternal care.

The rapid advancement of artificial intelligence (AI) has created new opportunities for healthcare. Large language models (LLMs) are capable of processing complex medical information, generating human-like responses, and supporting reasoning across clinical tasks (4). In nursing, their potential extends beyond clinical decision support to improving health literacy, strengthening patient education, and empowering self-care. Such applications are particularly valuable for pregnant and postpartum women at risk of VTE, who require ongoing guidance and support. Nevertheless, concerns persist about the accuracy, reliability, and safety of AI-generated outputs, especially in high-risk maternal contexts (5). Currently, several representative LLMs are in widespread use or rapid development. International models such as GPT-4.1 (OpenAI) and Claude 3.7 (Anthropic) are recognized for their strong comprehension and generalization capabilities. In China, DeepSeek and Kimi have shown rapid progress, while Huatuo has been optimized for the medical domain through training with clinical guidelines and literature (6–8). Despite growing popularity, systematic evaluations of these models in maternal health and nursing care remain limited (9).

To address this gap, the present study developed an expert-informed evaluation framework focused on the nursing management of maternal VTE. Using this framework, we systematically assessed the performance of five representative LLMs—DeepSeek, GPT-4.1, Claude 3.7, Huatuo, and Kimi—across six clinical domains: etiology, diagnosis, treatment, prognostic assessment, home care, and prevention. By evaluating outputs in terms of accuracy, comprehensibility, logical coherence, reliability, and safety, this study provides novel insights into the relative performance patterns and limitations of LLMs in maternal VTE care. Importantly, these findings are intended to inform comparative understanding of model strengths and weaknesses in nursing-relevant domains, rather than to determine their clinical adequacy for independent nursing practice.

## 2 Materials and methods

### 2.1 Study design

The study was designed around the practical needs of pregnant and postpartum women diagnosed with VTE. The development of the framework was informed by the consultation process and patient education delivered at different stages of care (admission, hospitalization, discharge, and follow-up) for pregnant and postpartum women with VTE. In combination with clinical expertise and evidence from the literature, these inputs guided the preliminary construction of dimensions and items addressing common concerns of this patient group. Subsequently, a two-round Delphi survey involving 30 clinical obstetric and gynecologic experts was conducted, resulting in the final set of 41 items that served as the core content of the evaluation framework. These items comprised 3 related to etiology, 4 to diagnosis, 17 to treatment, 5 to prognostic assessment, 4 to home care, and 8 to prevention.

In the first round, 30 questionnaires were distributed and all 30 valid responses were returned (response rate = 100%). The second round achieved the same (response rate = 100%). The expert panel comprised a multidisciplinary team of professionals from clinical medicine and nursing. All participants held intermediate or higher professional titles (73.33% senior level), 83.33% had over 10 years of work experience, and all possessed at least a bachelor’s degree (30% held master’s or doctoral degrees). Experts were geographically distributed across six provinces—Fujian, Guizhou, Zhejiang, Shanxi, Sichuan, and Chongqing—ensuring broad representativeness and authority.

Both rounds were conducted anonymously. Experts rated each item’s importance and relevance using a five-point Likert scale. Following statistical analysis of the first-round results, detailed feedback—including mean scores, interquartile ranges, percentage agreement, and integrated revision suggestions—was provided to all experts. They were then invited to re-evaluate the revised items in the second round. Items were retained if the mean score exceeded 3.5, the coefficient of variation was < 0.25, and ≥ 70% of experts rated the item as “important” or “very important.” The authority coefficient (Cr) and Kendall’s W were used to assess expert reliability and consensus. Ultimately, 41 validated items were finalized, comprising 3 for etiology, 4 for diagnosis, 17 for treatment, 5 for prognostic assessment, 4 for home care, and 8 for prevention.

Based on these 41 items, we systematically evaluated the performance of five AI models—DeepSeek, GPT-4.1, Claude 3.7, Huatuo, and Kimi—in addressing clinical questions commonly encountered in the management of maternal VTE. Among the included models, DeepSeek, Kimi, and Huatuo were developed in China. Huatuo is a large language model specifically optimized for the medical domain. Its training incorporates extensive medical literature, clinical guidelines, and diagnostic pathways, with the aim of providing safe and reliable medical question-answering support. As internationally leading general-purpose large language models, GPT-4.1 and Claude 3.7, demonstrate exceptional language understanding and cross-task generalization, performing robustly across a wide range of natural language processing tasks.

### 2.2 Evaluation and scoring method

This study evaluated the performance of five AI models across 41 items encompassing six clinical domains. For each item, all models generated responses based on standardized prompts to ensure consistency in comparison. To minimize potential bias, the AI-generated responses were anonymized and their presentation order was randomized prior to evaluation. An expert panel consisting of three obstetricians and gynecologists with extensive clinical experience and expertise independently assessed all responses. The evaluation was conducted across five key dimensions—accuracy, comprehensibility, logical coherence, reliability, and safety—using a five-point Likert scale. The expert ratings were used as a structured evaluation instrument to facilitate standardized comparison across models, rather than as a benchmark against human nurse or physician performance.

### 2.3 Statistical methods

All statistical analyses were conducted using SPSS version 29.0, and data visualization and graphical representations were generated with GraphPad Prism version 10.0. Fleiss’s Kappa coefficient was used to evaluate the inter-rater reliability among the three raters. This study used the Dunn *post hoc* test to compare the overall score differences of the five AI models in terms of accuracy, comprehensibility, logicality, reliability and security. Additionally, to assess the overall differences among the AI models within the six clinical domains, the Kruskal–Wallis H test was applied for each dimension. To control for type I error due to multiple comparisons, all pairwise *p* values were adjusted using the Bonferroni correction. Adjusted *p* < 0.05 were considered statistically significant. Composite domain-level scores used in the heatmap were calculated as the unweighted mean of the five evaluation dimensions.

## 3 Results

### 3.1 Inter-rater reliability

Fleiss’s Kappa analysis indicated excellent inter-rater agreement among the three evaluators (Kappa = 0.892, *Z* = 61.579). Pairwise comparisons further confirmed high consistency: evaluator 1 vs. evaluator 2 (Kappa = 0.861, *Z* = 33.351), evaluator 1 vs. evaluator 3 (Kappa = 0.894, *Z* = 34.560), and evaluator 2 vs. evaluator 3 (Kappa = 0.944, *Z* = 36.470). All weighted Kappa values were statistically significant (*p* < 0.001) and, according to conventional benchmarks, each exceeded the threshold for the excellent range.

### 3.2 Comparative evaluation across five dimensions

**Figure 1:**
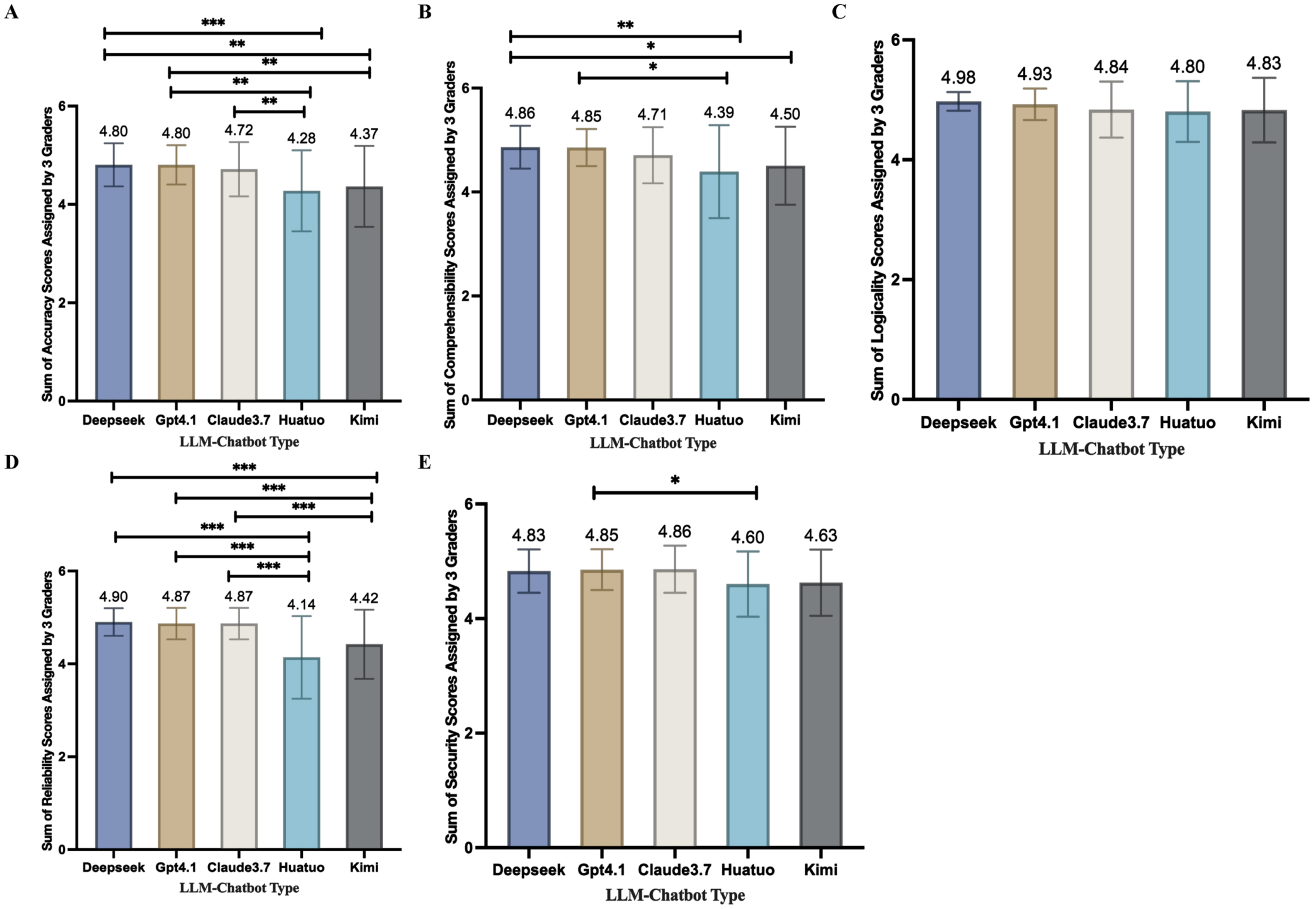
Three obstetrics and gynecology experts rated the responses from DeepSeek, GPT-4.1, Claude 3.7, Huatuo, and Kimi across five dimensions: overall accuracy, comprehensibility, logical coherence, reliability, and safety. **(A)** Average overall accuracy scores of the five AI models. **(B)** Average overall comprehensibility scores of the five AI models. **(C)** Average overall logicality scores of the five AI models. **(D)** Average overall reliability scores of the five AI models. **(E)** Average overall security scores of the five AI models. *indicates *p* < 0.05, **indicates *p* < 0.01, and ***indicates *p* < 0.001. Statistical significance was determined using Dunn’s post hoc test with Bonferroni correction for multiple comparisons.

As shown in Figure 1, in terms of interpretability, Huatuo achieved a lower mean score (4.39 ± 0.89) compared with DeepSeek (4.86 ± 0.41; *p* = 0.003) and GPT-4.1 (4.85 ± 0.36; *p* = 0.011). Kimi also showed a lower mean interpretability score (4.50 ± 0.75) than DeepSeek (4.86 ± 0.41; *p* = 0.037). No significant differences were observed across models with respect to logical coherence. Regarding reliability, Huatuo obtained a significantly lower mean score (4.14 ± 0.89) than DeepSeek (4.90 ± 0.30; *p* < 0.001), GPT-4.1 (4.87 ± 0.34; *p* < 0.001), and Claude 3.7 (4.87 ± 0.34; *p* < 0.001). Kimi likewise demonstrated a significantly lower reliability score (4.42 ± 0.75) compared with all three models (all *p* < 0.001). For safety scores, Huatuo yielded a lower mean score (4.60 ± 0.57) relative to Claude 3.7 (4.86 ± 0.41; *p* < 0.037). Although several differences reached statistical significance, the absolute mean differences across models were generally modest, typically ranging from approximately 0.2–0.4 points on the 5-point scale, indicating small effect sizes.

### 3.3 Model performance in six clinical domains

**Figure 2:**
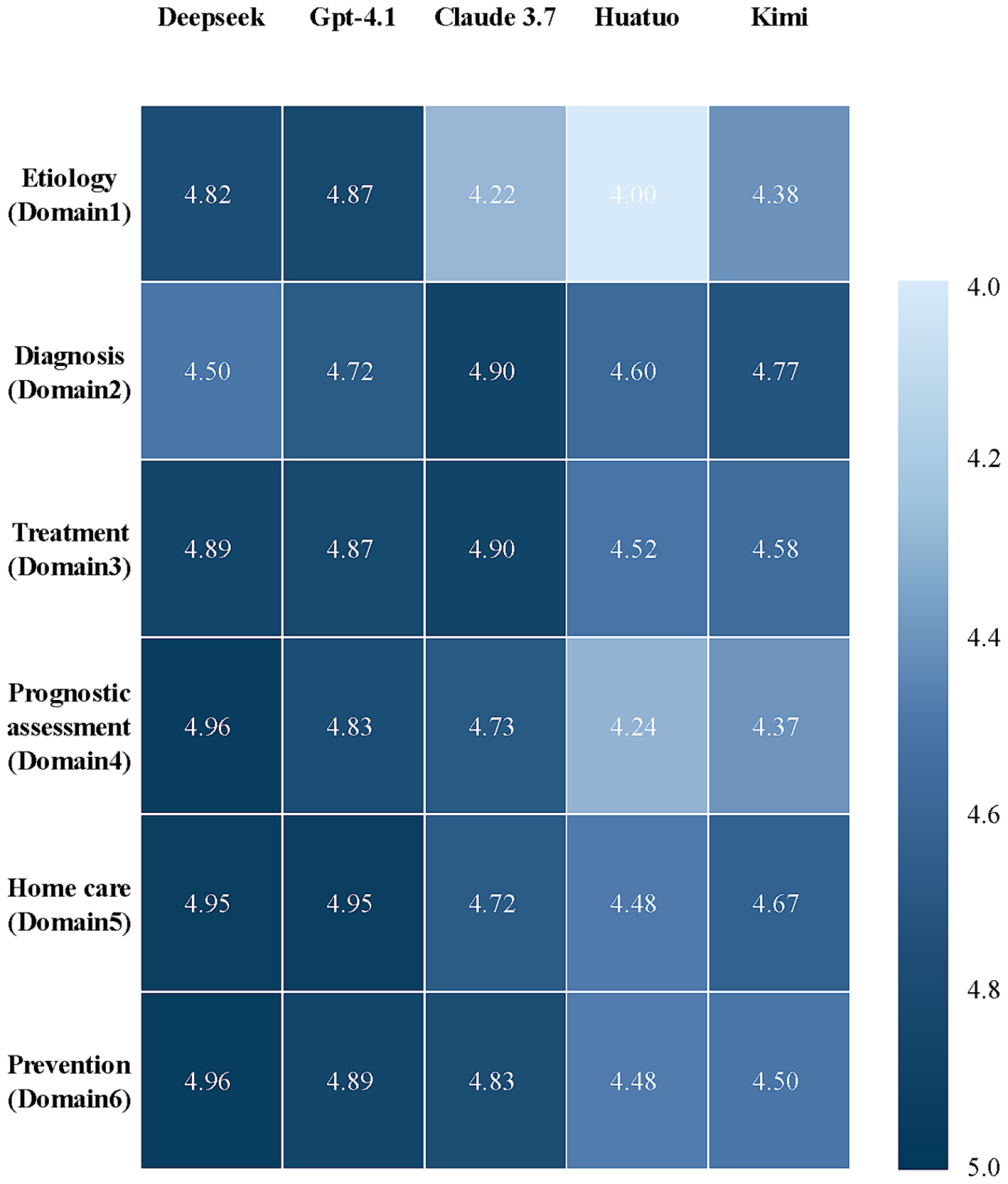
Integrative heatmap summarizing composite performance scores of five large language models across six clinical domains in maternal VTE care. Each cell represents the mean score aggregated across five evaluation dimensions (accuracy, comprehensibility, logical coherence, reliability, and safety) within the corresponding clinical domain. Color intensity reflects relative performance levels, with darker shades indicating higher composite scores.

**Figure 3:**
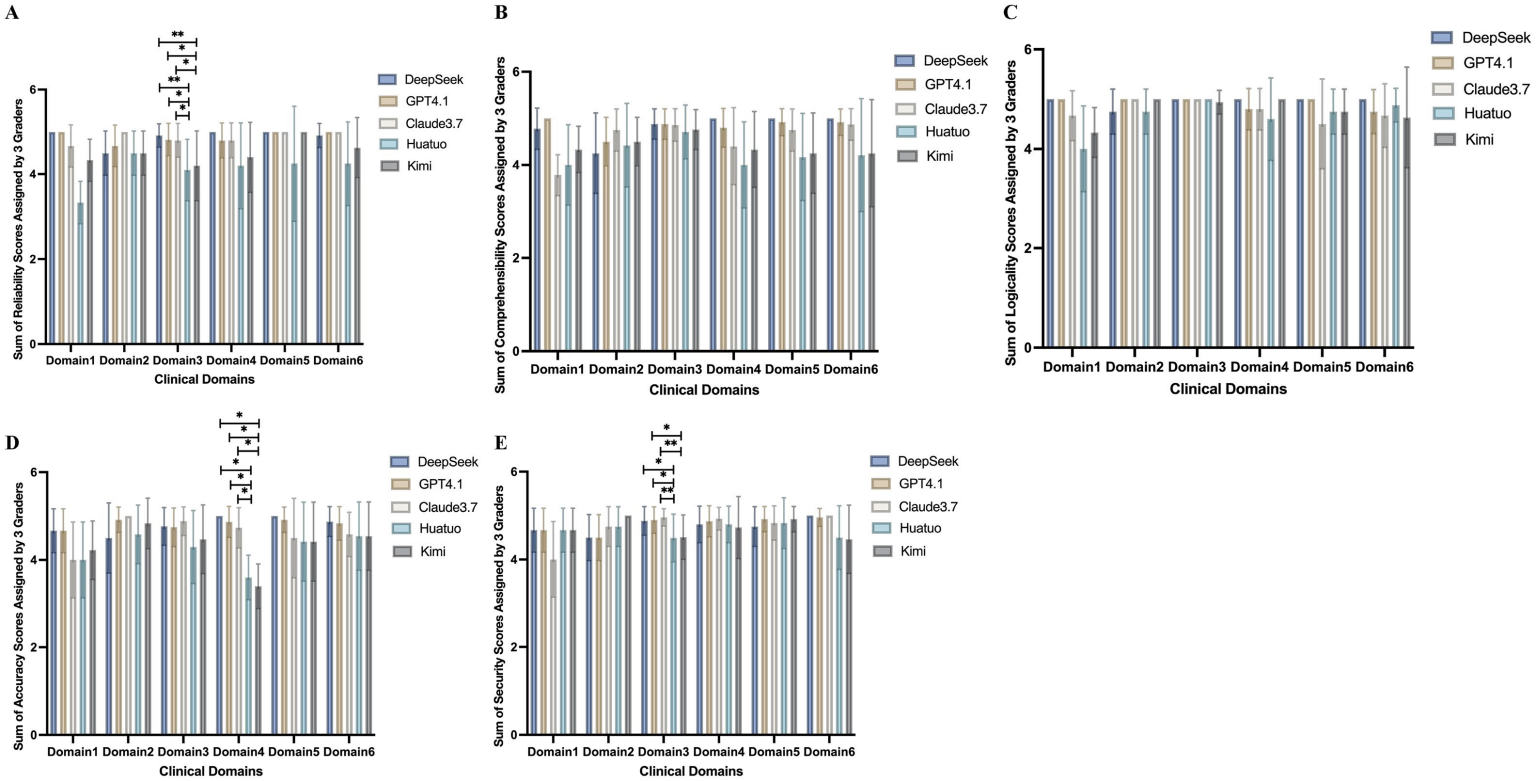
Performance of DeepSeek, GPT-4.1, Claude 3.7, Huatuo, and Kimi across six clinical domains in maternal VTE. **(A)** Average overall accuracy scores of the five AI models across the six clinical domains. **(B)** Average overall comprehensibility scores of the five AI models across the six clinical domains. **(C)** Average overall logical coherence scores of the five AI models across the six clinical domains. **(D)** Average overall reliability scores of the five AI models across the six clinical domains. **(E)** Average overall safety scores of the five AI models across the six clinical domains. *indicates *p* < 0.05 and **indicates *p* < 0.01. Statistical significance was determined using Dunn’s post hoc test with Bonferroni correction for multiple comparisons.

To facilitate interpretation of the multidimensional comparison structure, an integrative heatmap summarizing mean performance patterns across models and clinical domains is presented in Figure 2. The composite score in the heatmap represents the unweighted mean of the five evaluation dimensions (accuracy, comprehensibility, logical coherence, reliability, and safety) within each clinical domain. The heatmap highlights overall performance gradients, while detailed statistical comparisons are presented in Figure 3. As illustrated in Figure 3, we evaluated the performance of five AI models across six clinical domains commonly encountered in patients with maternal VTE. With respect to accuracy, no significant differences were observed among the models in the domains of etiology, diagnosis, treatment, home care, and prevention. However, in the domain of prognostic assessment, Huatuo demonstrated significantly lower scores compared with DeepSeek, GPT-4.1, and Claude 3.7 (all *p* = 0.014), while Kimi also showed significantly lower scores than these three models (all *p* = 0.031). Notably, these statistically significant differences were associated with relatively small absolute score differences, suggesting modest effect sizes within the context of the 5-point rating scale. For comprehensibility and logical coherence, no statistically significant differences were observed across the six domains. In terms of reliability, differences emerged in the treatment domain. Huatuo scored lower than DeepSeek (*p* = 0.033) and Claude 3.7 (*p* = 0.033), and significantly lower than GPT-4.1 (*p* = 0.003). Similarly, Kimi scored lower than DeepSeek (*p* = 0.044) and Claude 3.7 (*p* = 0.044), and significantly lower than GPT-4.1 (*p* = 0.005). Regarding safety, significant differences were also confined to the treatment domain. Huatuo scored lower than DeepSeek (*p* = 0.047) and GPT-4.1 (*p* = 0.013), and significantly lower than Claude 3.7 (*p* = 0.003). Kimi likewise scored lower than DeepSeek (*p* = 0.018) and Claude 3.7 (*p* = 0.004).

## 4 Discussion

Our study systematically evaluated the performance of five large language models—DeepSeek, GPT-4.1, Claude 3.7, Huatuo, and Kimi—within the nursing context of maternal VTE. The results showed that international general-purpose models (GPT-4.1 and Claude 3.7) and the domestic general model (DeepSeek) performed strongly in terms of accuracy, reliability, and safety, while Huatuo and Kimi were less reliable in high-risk domains such as treatment and prognostic assessment. The high inter-rater reliability (Kappa = 0.892) indicates that the evaluation framework applied in this study is both robust and feasible. Compared with previous research, which primarily focused on medical diagnosis, surgical decision-making, or oncology (10–12), our study emphasizes the underexplored field of maternal nursing. The overall findings align with prior studies showing the superiority of GPT-4 models across multiple dimensions (13) and Claude’s advantage in clarity and patient-centered communication (14). Importantly, this study underscores the nursing value of LLMs, particularly in education, home care, and preventive guidance (15, 16).

Our study bridges nursing practice and artificial intelligence by establishing a Delphi-based, evidence-informed evaluation framework that systematically connects LLM performance metrics with nursing decision-making and patient education needs. From a nursing perspective, LLMs offer multifaceted value in the management of maternal VTE. First, in patient education and health promotion, Claude 3.7 and GPT-4.1 demonstrated superior comprehensibility and logical coherence, enabling nurses to explain disease risks, anticoagulation precautions, and post-discharge home care more clearly to pregnant and postpartum women and their families, thereby enhancing health literacy and treatment adherence (17). Second, in the development of nursing care plans, LLMs provided relatively reliable information on etiology, diagnosis, and preventive strategies, which can assist nurses in designing individualized interventions (18). Third, in follow-up and remote nursing care, LLMs have the potential to provide timely informational support, partially alleviating the shortage of nursing resources, particularly in primary healthcare institutions or settings with limited nursing staff (19). It is important to emphasize that the present study does not assess whether any LLM meets an acceptable threshold for independent nursing practice. The findings reflect relative differences in model performance under a predefined expert-informed framework. Without direct comparison to human nurse or physician performance, conclusions regarding clinical adequacy or readiness for real-world nursing deployment cannot be drawn from the current design. It is important to note that several statistically significant differences observed in this study were associated with relatively small absolute score differences on a 5-point rating scale. While such differences may reach statistical significance due to consistent expert ratings, their practical relevance for nursing decision support should be interpreted with caution. In this context, small effect sizes may reflect incremental improvements in clarity, reliability, or safety rather than substantively different clinical guidance. Therefore, these findings are best understood as indicators of relative performance patterns among models, rather than as evidence of meaningful clinical superiority or readiness for independent nursing decision-making. Nevertheless, this study also revealed that Huatuo and Kimi performed notably worse in high-risk domains such as treatment and prognostic assessment. This finding is highly consistent with the sensitivity of high-stakes decision-making in nursing practice, where inappropriate recommendations may increase patient risk (20). Consequently, the application of LLMs in nursing should be positioned as a supplementary tool rather than a substitute for professional judgment. Nurses must exercise critical thinking, integrate evidence-based guidelines with clinical experience, and rigorously evaluate the scientific validity and safety of AI-generated outputs (21, 22).

It is noteworthy that domestic models demonstrated considerable potential in this study. Among them, DeepSeek performed at a level approaching that of leading international models, suggesting strong feasibility for application in nursing contexts (23). Compared with international models, one of DeepSeek’s key advantages lies in its ability to support localized deployment. This feature is particularly important in healthcare and nursing: on the one hand, localized operation ensures compliance with national regulations and institutional policies regarding patient privacy and data security (24, 25); on the other hand, it facilitates integration with in-hospital electronic medical records and nursing information systems, thereby improving responsiveness and practicality (26). Consequently, DeepSeek may gradually gain a competitive edge in future nursing and healthcare practice due to its compliance and operational advantages. Nevertheless, challenges remain in the development of domestic models. A certain degree of “involution” exists, with resources disproportionately concentrated on leading general-purpose models, while domain-specific or emerging products such as Huatuo and Kimi risk being marginalized if they lack distinctive competitive advantages (27, 28). In practice, hospitals are unlikely to deploy multiple large models simultaneously and will ultimately favor a small number of systems with comprehensive advantages—further reinforcing DeepSeek’s potential dominance. At the international level, although GPT-4.1 and Claude do not currently offer localized deployment, they continue to hold a leading position due to access to broader international medical and nursing datasets, stronger cross-domain reasoning capabilities, and adaptability across diverse contexts (29). In clinical and nursing applications across Europe and North America, these models can be continuously optimized through real-world data, strengthening their global competitiveness (30). Taken together, these dynamics suggest the emergence of a regionalized landscape: DeepSeek may maintain an advantage in the Chinese healthcare and nursing context through localization and compliance, whereas GPT-4.1 and Claude are likely to retain global leadership by leveraging international resources and robust ecosystems. This trend highlights the importance for nursing science to carefully consider the adaptability and sustainability of different models, thereby achieving complementarity between localized and international applications (31, 32).

This study has several limitations. First, the expert sample size was relatively small, with only three nursing professionals involved in the final evaluation phase, and the evaluators were drawn from a limited geographic and institutional context within six provinces in China. Although high inter-rater reliability was observed, this expert sample may not fully represent the diversity of clinical practices, institutional settings, or educational backgrounds across different regions. As a result, potential selection bias cannot be entirely excluded, and the generalizability of the findings may be limited. Second, the evaluation focused exclusively on maternal VTE, and only text-based interactions were assessed. Multimodal inputs and real-world clinical deployment were not examined in the present study. Future research should incorporate larger and more diverse panels of nursing experts across multiple institutions and geographic regions, as well as real clinical environments, to enhance representativeness, external validity, and practical applicability. In the future, integrating validated LLMs into nursing informatics systems and mobile health platforms may facilitate real-time, context-aware clinical decision support and personalized maternal education.

## 5 Conclusion

This study systematically compared five LLMs in the nursing context of maternal VTE. GPT-4.1, Claude 3.7, and DeepSeek showed strong overall performance, while Huatuo and Kimi demonstrated weaknesses in high-risk domains. LLMs demonstrate relative strengths in nursing-relevant domains such as patient education and follow-up when compared under a standardized framework; however, their clinical adequacy for independent nursing practice requires further validation.

## Data Availability

The original contributions presented in the study are included in the article/supplementary material, further inquiries can be directed to the corresponding author.
